# Artificial Proteins Designed from G3LEA Contribute to Enhancement of Oxidation Tolerance in *E. coli* in a Chaperone-like Manner

**DOI:** 10.3390/antiox12061147

**Published:** 2023-05-24

**Authors:** Jiahui Han, Shijie Jiang, Zhengfu Zhou, Min Lin, Jin Wang

**Affiliations:** 1 Key Laboratory of Agricultural Microbiome (MARA), Biotechnology Research Institute, Chinese Academy of Agricultural Sciences, Beijing 100081, China; 2 School of Life Science and Engineering, Southwest University of Science and Technology, Mianyang 621010, China

**Keywords:** 11-mer motifs, artificial proteins, oxidation tolerance, enzyme activity, chaperone-like role

## Abstract

G3LEA is a family of proteins that exhibit chaperone-like activity when under distinct stress. In previous research, DosH was identified as a G3LEA protein from model extremophile—*Deinococcus radiodurans* R1 with a crucial core HD domain consisting of eight 11-mer motifs. However, the roles of motifs participating in the process of resistance to stress and their underlying mechanisms remain unclear. Here, eight different proteins with tandem repeats of the same motif were synthesized, named Motif1–8, respectively, whose function and structure were discussed. In this way, the role of each motif in the HD domain can be comprehensively analyzed, which can help in finding possibly crucial amino acid sites. Circular dichroism results showed that all proteins were intrinsically ordered in phosphate buffer, and changed into more α-helical ordered structures with the addition of trifluoroethanol and glycerol. Transformants expressing artificial proteins had significantly higher stress resistance to oxidation, desiccation, salinity and freezing compared with the control group; *E. coli* with Motif1 and Motif8 had more outstanding performance in particular. Moreover, enzymes and membrane protein protection viability suggested that Motif1 and Motif8 had more positive influences on various molecules, demonstrating a protective role in a chaperone-like manner. Based on these results, the artificial proteins synthesized according to the rule of 11-mer motifs have a similar function to wildtype protein. Regarding the sequence in all motifs, there are more amino acids to produce H bonds and α-helices, and more amino acids to promote interaction between proteins in Motif1 and Motif8; in addition, considering linkers, there are possibly more amino acids forming α-helix and binding substrates in these two proteins, which potentially provides some ideas for us to design potential ideal stress-response elements for synthetic biology. Therefore, the amino acid composition of the 11-mer motif and linker is likely responsible for its biological function.

## 1. Introduction

LEA proteins are a class of proteins that are involved in growth, development and abiotic stress tolerance [1,2]. Currently, group 3 LEA proteins (G3LEA) are the most extensively studied. G3LEA proteins are characterized by high hydrophilicity and low sequence complexity [3], which play roles in stabilizing the cell membrane, molecular barriers and anti–oxidation under abiotic stress [4]. Investigations into the secondary structure of G3LEA proteins revealed that the majority of G3LEA proteins were predominantly disordered under normal condition [5,6,7,8] and converted into an ordered structure upon desiccation [9]. As previously reported, these proteins were capable of improving the host resistance with heterologous expression and had a stabilizing effect on enzymes in vitro [10]. DosH was identified as a G3LEA protein [10] and characterized by a naturally ordered structure which was not same for those of other G3LEA proteins [11].

There is a key region in DosH, named HD (Hydrophilic Domain) [10,11]. HD contained the same proportion of helical structure after structural transitions compared with other HDs of G3LEA, but *E. coli*, expressing the HD protein (8 motifs), had a higher survival rate than those expressing the CeHD (from *Caenorhabditis elegans*, 24 motifs) and YlHD (*Yarrowia lipolytica*, 27 motifs) under both desiccation and oxidation conditions. Additionally, MST (Microscale Thermophoresis) results illustrated that the HD/LDH (Lactate dehydrogenase) pair displayed the strongest interaction. The results of LDH aggregation inhibition and protection indicated that HD was capable of exhibiting stronger enzyme activity protection [10], showing that HD acted in a chaperon-like manner. Based on these results, the HD showed promise as an ideal stress-response element in synthetic biology [10]. HD was composed of eight 11-mer motifs, which is in accordance with the typical characteristic of G3LEA. Additionally, the consensus of motifs is “ΦΦΩXΦΨΩΨΦXΩ”, where Φ/Ω/Ψ represent hydrophobic residues, negatively charged or amide residues, and positively charged residues, respectively, and X represents a nonspecifically conserved amino acid residue [12,13,14].

It was recently reported that several model peptides of G3LEA protein, such as PvLEA-22, which consists of two tandem repeats of the 11-mer motif, PvLEA-44, which was made of four tandem repeats of the same 11-mer motif, and the scrambled peptides sequences composed of amino acids same with motif, were established using synthetic biology to investigate their protective effect on enzymes under stress conditions. The results indicated that, except the randomized amino acid sequence, all of the G3LEA models transformed from random coils into α-helix upon dehydration and returned to random coils again upon rehydration. Moreover, the degree of protection was comparable to that of full-length G3LEA proteins [14,15,16,17].

This strategy showed us, on one hand, whether the sequences characterized by specific rule 11-mer amino acids might have similar functions and if there are more amino acids contributing to binding substrates or α-helix formation, which will be changed in function, and on the other hand, whether we could synthesize a series of proteins made of eight tandem repeats of same 11-mer motifs of HD in order, respectively. In this study, we synthesized eight proteins that were naturally ordered, possessed chaperone-like features and are potentially involved in *E. coli* stress tolerance. There are different amino acids in different motifs and linkers, leading to a difference in Motif function. In addition, we described the extraordinary functions of Motif1 and Motif8, which are better than other artificial proteins. According to the amino acids contained in the motifs and linkers, we also proposed the best scheme for designing artificial proteins with chaperone-like functions as a reference.

## 2. Results

### 2.1. Sequence Composition within the HD Domain

**Figure 1:**
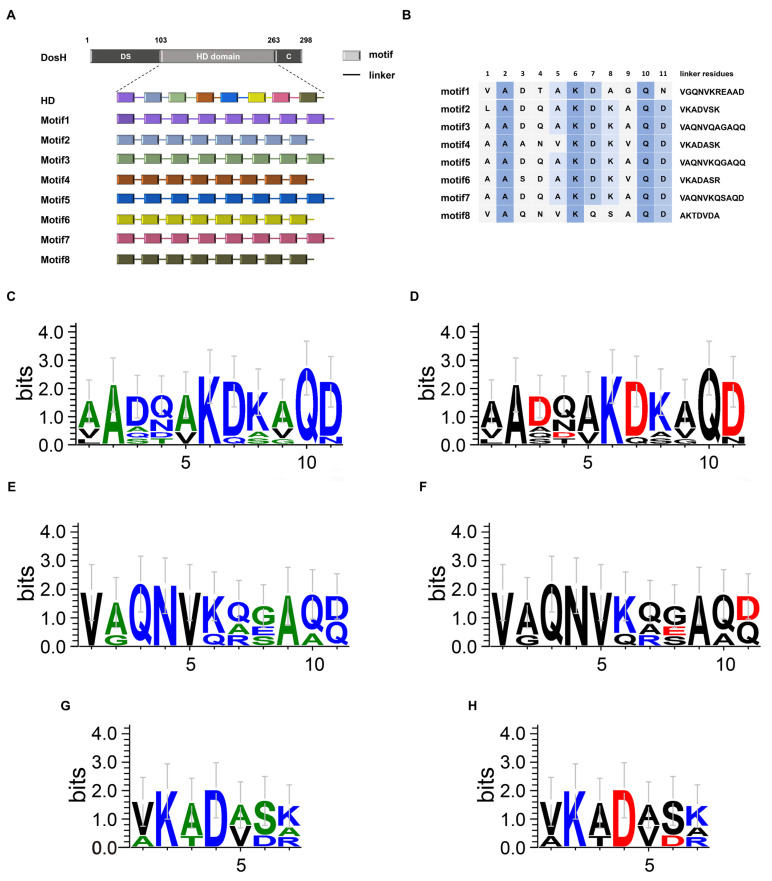
The model and sequence comparison of eight artificial proteins with tandem repeating motifs and linkers. (**A**) The schematic of construction of eight artificial proteins and (**B**) amino acid distribution in every motif and linker. The comparison of motifs with hydrophobicity (**C**) and charge properties (**D**), respectively. The comparison of long linkers (11 amino acids) with hydrophobicity (**E**) and charge properties (**F**), respectively. The comparison of short linkers (7 amino acids) with hydrophobicity (**G**) and charge properties (**H**), respectively.

The protein DosH from *Deinococcus radiodurans* R1 contains 298 amino acids and is divided into three regions: the DS domain (position from 1 to 103), hydrophilic domain (HD) (position from 104 to 263) and C–terminal (position from 264 to 298). The HD domain contains eight conserved but different motifs. Protein Motif1 composes eight first motifs and linkers in series; protein Motif2 composes eight second motifs and linkers in series, and the rest may be deduced using an analogy (Figure 1A). Motif1, Motif3, Motif5 and Motif7 contain 176 amino acids; Motif2, Motif4, Motif6 and Motif8 contain 144 amino acids. These eight proteins are all hydrophilic according to the results shown in a study by Kyte and Doolittle (Supplementary Figure S2).

The sequences of each motif and linker are shown in Figure 1B. The more conserved the amino acid site, the darker its background color. Each motif almost conforms to the rule of 11-mer motifs and the amino acids at positions 2, 6 and 10 are the most conserved. The “KDK” conserved sequence exists in most HD motifs, but it does not seem to follow this rule completely in Motif1 and Motif8. In Motif1, “A” in position 8 replaces the second “K” and “N” in position 11 replaces “D”, and in Motif8, “Q” and “S” in position 7 and 8 replace “D” and “K”, respectively. There are some differences in position 1; “L” in Motif2 and “V” in Motif1 and Motif8, but they belong to hydrophobic amino acids. Regarding the linkers, there are 11 residues in Motif1/3/5/7 and some differences in amino acids at several sites. “V” in position 1, “A” in position 9 and “QNV” in positions 3, 4 and 5 are absolutely conserved in four linkers. There is “R” in position 7, “E” in position 8 and “A” in position 10 of linker 1, “S” in position 8 of linker 7, which is maybe the greatest difference from others. There were seven residues in Motif2/4/6/8, including “T” and “A” in Motif8, which differs from the corresponding sites in other linker sequences. Some other sites may not be completely conserved, but are consistent in being charged or hydrophobic. In Figure 1C,D, we compared the eight motifs with hydrophobicity and charge properties using WebLogo3, respectively. The results showed that, as mentioned above, the “KDK” sequence was relatively conserved. Furthermore, the amino acids characteristics at each site are fully exhibited in these two figures. We also performed amino acid alignments regarding linkers containing 11 (Figure 1E,F) and 7 (Figure 1G,H) amino acids with hydrophobicity and charge properties, respectively.

### 2.2. CD Analysis of Artificial Hydrophilic Proteins

Previous studies have shown that G3LEA proteins were disordered under natural conditions, and would transform into ordered structures under drying, glycerol and/or SDS conditions [10,17,18,19]. Similarly, the HD was disordered under phosphate buffer and transformed into an ordered structure under 50% glycerol and 50% TFE [10,11]. Therefore, we speculate that eight proteins with tandem repeating motifs also possess natural disordered structures. Henceforth, to confirm the above conjecture, *E. coli* BL21 (DE3) cells were transformed with pET28a (+) plasmid encoding genes of nine proteins (molecular weight was 21.2, 38.9, 36.3, 38.9, 35.7, 39.2, 35.7, 39.3 and 36.8 kD, respectively) (Supplementary Figure S1) and induced to a final concentration of 0.3 mM at 16 °C using IPTG. The proteins were analyzed using far-UV circular dichroism (CD) spectroscopy with purification and concentration. If the CD spectrum has a maximum peak at 192 nm and a minimum peak at 208 and 222 nm, this curve shape indicates that the proteins mainly contain α-helices structure. If the CD spectrum has a minimum peak near 216 nm and a maximum peak between 185–200 nm, this curve shape indicates that the proteins mainly contain β-sheet structure. If the CD spectrum has a maximum peak around 206 nm, this curve shape indicates that the proteins mainly contain β-turn structure. If the CD spectrum has a negative peak near 198 nm and a small and wide peak around 220 nm, this curve shape indicates that the proteins mainly contain random coils. The curves with similar shape differ in peak value or curve intensity indicates that the content of the main secondary structure of the protein is different.

**Figure 2:**
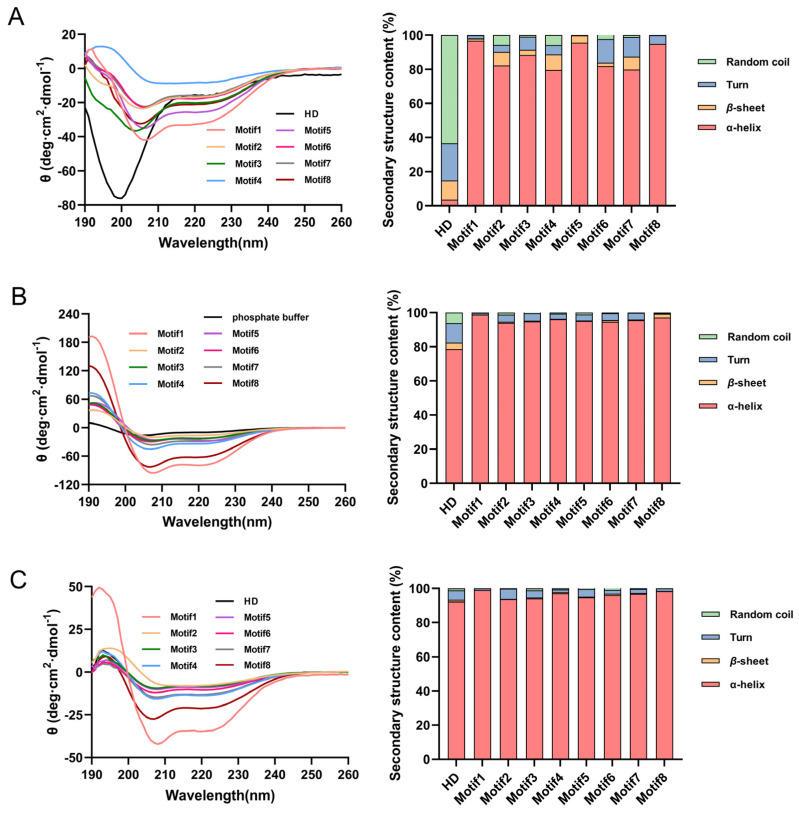
Changes in far-ultraviolet (UV) circular dichroism (CD) spectra of HD and eight artificial proteins. The structural transformation of proteins under 50% TFE (**B**) and 50% glycerol (**C**) compared with phosphate buffer (**A**).

Interestingly, the UV absorption of HD protein had a negative peak at 198 nm and a small, wide peak around 220 nm, which was different from those of artificial proteins (Motif1–8) forming their maximum peak at approximately 192 nm and minimum peaks at 208 and 222 nm, indicating that these artificial proteins were ordered under phosphate buffer (Figure 2A). The stacked bar graphs in the right half of Figure 2 showed that the α-helix content of proteins became greater than previously with the addition of 50% TFE (as a well-known α-helix inducer in general) or 50% glycerol (generally used to mimic waterloss conditions) (Supplementary Table S2) [20,21,22,23,24], implying that, the HD protein had undergone steric modification with the process of changing from a disordered to ordered structure. Conversely, the artificial proteins showed a big increase in spectrum at 192 nm, 208 nm and 222 nm, and their second structures were more ordered (Figure 2B,C). Meanwhile, we could find that the intensity of the CD spectrum curve of artificial proteins at 192 nm, 208 nm and 222 nm was different, indicating that artificial proteins had different content of α-helices structure. The corresponding contents of the secondary structures of the different proteins were exhibited in the stacked bar graphs in the right half of Figure 2 (The detailed values in Supplementary Table S2), showing that there were more α-helices in Motif1 and Motif8 with the addition of TFE and glycerol.

### 2.3. Artificial Proteins Conferred E. coli Tolerance to Oxidation, Desiccation, Salinity and Freezing

**Figure 3:**
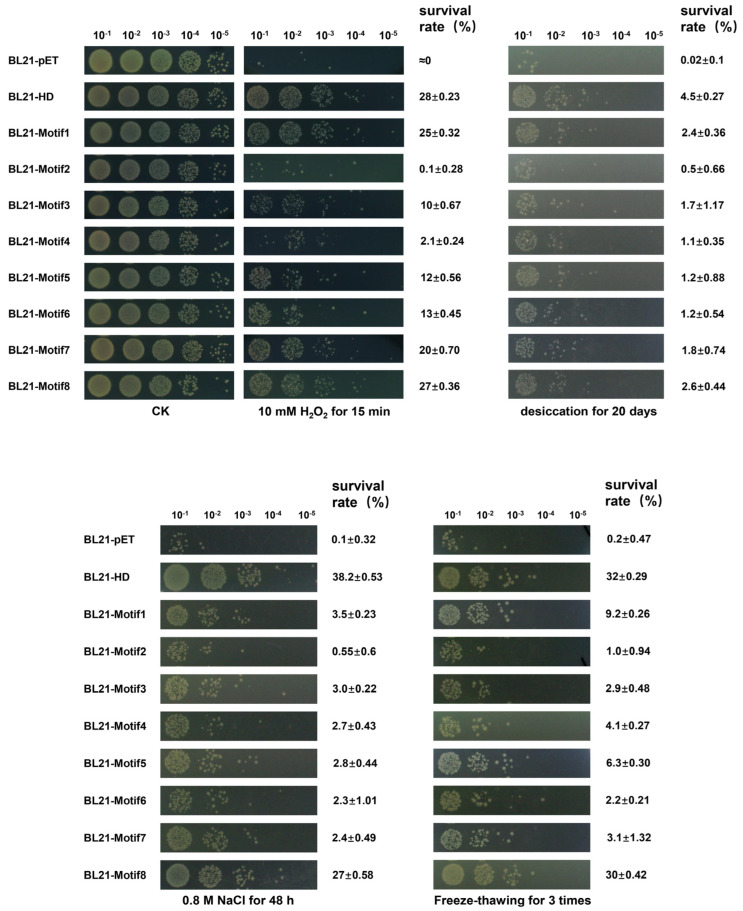
Survival phenotypic analysis of *E. coli* recombinant strains under different conditions. The strains were treated by 10 mM H_2_O_2_ for 15 min, desiccation for 20 days, 0.8 M NaCl for 48 h and freeze-thawing for three times for 20 min each time. Spotted agar plates after different treatments and serial dilution. BL21–pET, recombinant strain carried an empty pET28a vector, BL21–HD, recombinant strain expressing HD protein, BL21–Motif1, recombinant strain expressing Motif1 protein, BL21–Motif2, recombinant strain expressing Motif2 protein, BL21–Motif3, recombinant strain expressing Motif3 protein, BL21–Motif4, recombinant strain expressing Motif4 protein, BL21–Motif5, recombinant strain expressing Motif5 protein, BL21–Motif6, recombinant strain expressing Motif6 protein, BL21–Motif7, recombinant strain expressing Motif7 protein, BL21–Motif8, recombinant strain expressing Motif8 protein.

The nine *E. coli* transformants of overexpressed genes (*HD*, *Motif1*, *Motif2*, *Motif3*, *Motif4*, *Motif5*, *Motif6*, *Motif7* and *Motif8*) under different abiotic stresses containing H_2_O_2_ (10 mM for 15 min), desiccation (for 20 days), NaCl (0.8 M for 48 h) and freeze–thawing (20 min, 3 times), were placed onto LB agar medium. The results showed that, compared to the control (containing pET28a vector only), nine transformants indeed displayed higher oxidation resistance after treated with H_2_O_2_, and in terms of their survival rate, the strains BL21–Motif1 and BL21–Motif8 had the strongest viability, and BL21–Motif2 was the weakest. Similarly, the survival rate of nine transformants was as follows: BL21–HD > BL21–Motif8 ≈ BL21–Motif1 > BL21–Motif7 > BL21–Motif5 > BL21–Motif6 > BL21–Motif3 > BL21–Motif4 > BL21–Motif2 under desiccation, NaCl and freeze–thawing stresses, which seemingly conformed to the same trend in oxidation (Figure 3). In summary, for artificial proteins, Motif1 and Motif8 contributed most to *E. coli* tolerance. In addition, the survival rates of cells at different stages are presented in Supplementary Figure S3.

### 2.4. Artificial Proteins Contributed to Higher Stabilization of the LDH/MDH Activity under Stress Conditions

In previous reports, it was mentioned that the HD could perform stabilizing enzyme activities with chaperone-like functions on LDH under oxidation and desiccation conditions in vitro [10]. Therefore, the enzyme activity with HD was used as a control. In order to test protective ability of eight artificial proteins, the LDH and MDH enzymes were used as a reporter enzyme that were commonly used for an enzymatic activity protection assay [25,26,27], as well as BSA serving as the positive control.

**Figure 4:**
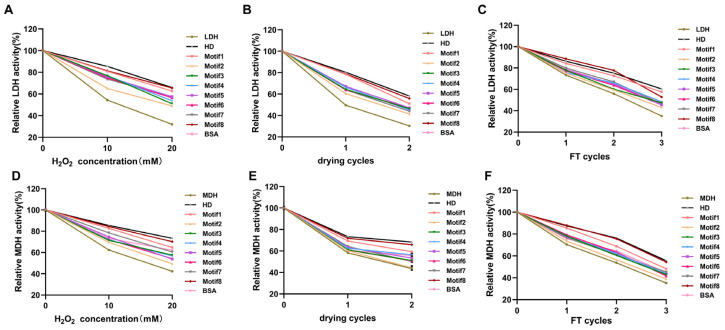
The relative activity of LDH/MDH under the oxidation, desiccation and freezing treatments. The residual activity of LDH (**A**) and MDH (**D**) after 10/20 mM H_2_O_2_ for 15 min. The residual activity of LDH (**B**) and MDH (**E**) after repeated desiccation. The residual activity of LDH (**C**) and MDH (**F**) after repeated freeze–thawing. All data were calculated from three sets of independent experiments.

As shown in Figure 4A, the LDH activity suffered obvious damage in phosphate buffer after oxidation treatment. However, the LDH activity with proteins (proteins:LDH = 3:1) showed a 1.48-, 1.20-, 1.42-, 1.42-, 1.40-, 1.38-, 1.36-, 1.51-fold increase compared with the control (LDH in phosphate buffer, 50% activity retained), respectively (Figure 4A), suggesting that Motif1 and Motif8 exhibited the strongest protection ability. In a similar way, the addition of artificial proteins also increased LDH enzyme activities under other diverse abiotic stresses, including drying and freeze–thawing (Figure 4B,C).

Furthermore, for MDH activity, the stabilizing function of proteins under diverse abiotic stresses followed a similar trend (Figure 4D–F). The highest significant residual MDH activity after treatment was observed in samples containing protein Motif1 and Motif8, whereas Motif2 protection was the weakest.

The results demonstrated that among proteins, Motif1 and Motif8 performed better than other proteins. Therefore, one of the leading hypotheses is that motif1 and motif8 play primary roles in the HD. In addition, further experiments were conducted to examine the relationship between enzyme activity and protein amounts, and the results are shown in Supplementary Figure S4, indicating that enzyme activities are proportional to protein amounts.

### 2.5. Artificial Proteins Increased the Total Antioxidant Capacity of E. coli Transformants

**Figure 5:**
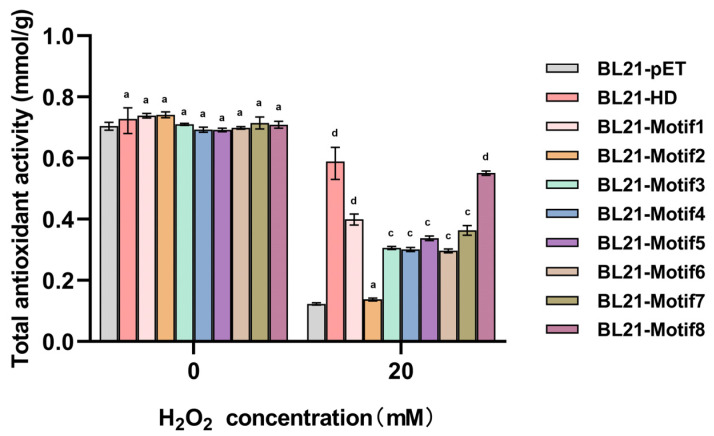
The total antioxidant activity of the transformants. All data were calculated from three sets of independent experiments. The mean ± standard error (SE) was used to show the data. The letters “a”, “c” and “d” represent *p* > 0.05, 0.001 < *p* ≤ 0.01, 0.0001 < *p* ≤ 0.001, respectively.

There is an antioxidant defense system equipped with enzymatic and non-enzymatic antioxidants in *E. coli* cells. Enzymatic antioxidants including CAT and other enzymes and non-enzyme systems including certain molecules reflect the total antioxidant capacity [28]. Together, antioxidants work to reduce the cell’s oxidative state. To further quantify the contribution of artificial proteins to cell resistance under oxidative stress, total antioxidant capacity was measured in different transformants (Figure 5). The results revealed that the total antioxidant capacity reduced after 20 mM H_2_O_2_ for 15 min in all strains compared to the untreated group, and that of artificial proteins followed a trend similar to LDH/MDH activity, which supported that the theory artificial proteins might act as oxidant scavengers under oxidative stress [29]. The strains expressing Motif1 and Motif8 proteins had a higher capacity than any other strains with artificial proteins (the expression levels of artificial proteins in Supplementary Figure S5). These data further supported that proteins Motif1 and Motif8 exhibited stronger protection ability and ROS scavenging activity.

### 2.6. E. coli Containing Artificial Proteins Had Higher CAT Activity and Less MDA Production

Aerobic organisms require oxygen molecules for ATP synthesis via oxidative respiration [30]. However, aerobic respiration generates reactive oxygen species (ROS) as by-products, mainly containing superoxide (O_2_^−^) or hydrogen peroxide (H_2_O_2_), occurring in many microbes, including *E. coli* [31]. These molecules have been known to cause functional and structural damage to cell components, which can lead to cell lysis and eventually cell death. Cells contain a variety of defense mechanisms to detoxify any adverse effects caused by an accumulation of ROS. Catalase enzymes are recognized as cellular defense mechanisms acting against ROS with scavenging H_2_O_2_. Thus, its functions help balance the amount of cellular ROS [30].

In order to further confirm the antioxidative effect of artificial proteins, the activities of CAT and MDA were evaluated in different transformants. CAT significantly reduces oxidative stress by further breaking down hydrogen peroxide into molecular oxygen [32]. In Table 1, the CAT activity of all recombinants was almost no difference under normal conditions but decreased after oxidation treatment (20 mM H_2_O_2_ for 15 min). Notably, the transformants with Motif1 and Motif8, BL21–Motif1 and BL21–Motif8, respectively, had fewer reductions in enzyme activity. Conversely, BL21–pET had the lowest enzyme activity.

At the same time, the production of MDA (as an indicator of oxidative stress and lipid peroxidation) was measured. The results showed that the MDA content of nine recombinant strains was higher than that of the untreated group. In our study, the level of MDA in BL21–pET was significantly higher in the treated group than that in the control group (Table 2). Similarly, the results showed that the transformants that expressed artificial proteins produced less MDA than those in BL21–pET, and strains containing Motif1 and Motif8 led to a significant decrease in the content of MDA. These results further confirmed the strongest antioxidation activity of proteins Motif1 and Motif8. All the above results suggested that non-natural proteins might offer protection when *E. coli* was in an oxidative condition.

## 3. Discussion

After decades of research, it is believed that G3LEA proteins could mitigate ROS damage and participate in environmental stress, such as drying [33,34,35,36], salinity [34,37], oxidation [35] and freezing [38]. More and more researches have indicated that G3LEA proteins not only existed in plants, but also in many prokaryotes acting in a chaperone-like manner [39,40], for example, DosH, which is characterized by an intrinsically ordered secondary structure from *D. radiodurans* R1.

It has been reported that the repeated region comprising 11-mer motifs, named HD, is the key functional part of G3LEA proteins [18,41], and the different number of motifs characterized different G3LEA proteins [10,42]. To further determine the function of motifs, many researchers have progressively conducted research by synthetic biology methods. Several model peptides comprising two or four repeats of one of those motifs were synthesized to form a stable glassy matrix upon dehydration, which acted as a thermal stabilizer of biological glasses [17]. The model peptides derived from G3LEA, the degree of protection of which was comparable to that conferred by wild-type proteins, as reported previously for LDH [41], had the ability to preserve liposomes in the dry state simultaneously [43]. In vivo, *E. coli* cells expressing G3LEA peptides showed a better survival rate under stress conditions [40]. Combining the present results with a previous study, it was evident that the synthetic G3LEA model peptides had the ability not only to be stable enough to be maintained under stressful inhabitation conditions but also to preserve the catalytic activity of enzymes in a chaperone-like manner in the stress state by preventing the protein’s aggregation [10]. Based on these previous findings and the present results, we hypothesize that short peptides composed of motifs can work as a cytoskeleton in dried cells to enable anhydrobiotic organisms to survive desiccation environments or act as molecular shield to increase the survivability under abiotic stress, playing a similar role to full-length G3LEA proteins.

We synthesized eight artificial polypeptides/proteins according to the consensus of the 11-mer amino acid sequence of G3LEA proteins. Protein sequence analysis revealed that all proteins were stable enough and hydrophilic (Supplementary Table S1). This is once again convincingly demonstrated in Supplementary Figure S2. The silico analysis revealed that these proteins exhibited disordered structures that were abundant with random coils, but it turned out to be the opposite when using circular dichroism (CD) spectrometry in phosphate buffer (Figure 2A). The artificial proteins had an ordered structure in the phosphate buffer, and whose structure became more ordered with the presence of TFE and glycerol (Figure 2B,C). The structure conversion implicated that there were more α-helices forming under water loss conditions. However, the HD protein exhibited a random coil in the phosphate buffer, which may be due to anti-cooperative behaviors, such as charge repulsion [44], steric effects [45] or hydrogen bond occupancy within α-helices [46] between different motifs. This is hard to explain clearly now, requiring more investigation. Nonetheless, proteins Motif1 and Motif8 had a higher degree of α-helix structure and conferred *E. coli* the strongest viability under stresses environments (Figure 3). However, the survival capability of strains expressing Motif1 and Motif8 was not as good as that of the strain with HD protein. We speculate that HD protein contains eight different 11-mer motifs indicating that it contained relatively intact structure compared to original DosH and more amino acids (11 amino acids) at the C-terminal which includes α-helix formation and contributes to substrate binding amino acids. In other words, only when eight motifs exist at the same time can it give play to the strongest capacity.

In previous research, there were many results confirming that the HD directly interact with LDH to protect its activity under stresses termed as a molecular shield or with chaperone-like activity [10,47]. Apparently, all eight artificial proteins reduced stress-induced enzyme inactivation to some extent. It was noteworthy that, BSA, a known protectant [37], was slightly less efficient at protecting the enzyme when compared to Motif1 and Motif8 proteins, which exhibited stronger capability in terms of protection of LDH, MDH and CAT (Figure 4 and Table 1). In different articles, there are numerous reports showing that under abiotic stress conditions, proteins from the G3LEA family exhibit a chaperone-like function by protecting enzymes from inactivation, presumably because there is a similar helical structure to the BSA protein. In addition, there are model peptides derived from G3LEA proteins that exhibit a similar protective function. Thus, it is entirely acceptable that artificial proteins derived from HD protein have similar protective functions. However, this protective behavior, which is not possessed by every protein, is not universal. In addition, Guo et al. proved that HD protein could decrease the content of MDA by 1.5 M NaCl for 4 h [11]. Additionally, in this study, HD protein still played the same role under oxidative stress (Table 2). Moreover, it is interesting that this function was shared among all eight artificial proteins, and strains containing Motif1 and Motif8 had minimum levels of MDA content, which indirectly reflected that the degree of cell membrane damage of these two strains was the lowest [11,48]. As is well known, the amphipathic helices formed by the proteins under stress conditions enable protein–membrane interactions through the hydrophobic face of the helices [49,50]. Based on these, we were more interested in the amino acids of Motif1 and Motif8.

The charge number and hydrophobicity of the protein are the main factors affecting the spatial structure [11]. During the process of protein folding, the hydrophobic force drives the polypeptide chain to the folded state, whereas hydrogen bonds, ion-pairs, disulfide bonds and van der Waals interactions define its shape and keep it from falling apart [51]. These non-covalent forces, which arise from inter–residue interactions among sequential neighbors as well as those far away in the sequence, cooperatively form and stabilize the native structure [52]. There is a preference for amino acid residues in medium- (within a distance of ±3 or ±4 residues) and long-range (more than 4 residues) contacts [52]. The residues, such as Leu, Ala, Glu and Gln, had medium-range contact and were found to be α-helix-forming, implying that these residues influenced the formation and stabilization of helices; in addition, Thr is more prevalent in the α-helix, Arg, Leu, Asn and Ser participate in protein–protein interaction and substrate binding [53]. The residues Val, Trp, Phe and Leu had high long-range contact, which showed that hydrophobic residues mainly influence the long-range contacts. On the contrary, Lys and Asp were oppositely charged interactions between neighboring residues [54,55]. Residue-wise analysis showed that Leu, Val and Ala were highly preferred in the membrane environment [56], which might be involved in the process of protecting the membrane and reducing lipid oxidation. Instead of typical “KDK”, the second “K” in Motif1 and “D” in Motif8 were replaced by “A” and “Q”, respectively, which can promote greater H bonds and helix generation. In addition, “S” in Motif8, belonging to binding residues, showed the effect of static electricity and hydrogen bonding on the affinity of the protein–protein complex as described in previous articles (Figure 1B) [56]. The results showed that the function of Motif2 was the weakest, but the amino acid residues of Motif2 conformed to the rule of the 11-mer motifs. Therefore, we turned to analysis of the linker sequences. Since the number of amino acids in the linker sequence for Motif2,4,6,8 was consistent, all having seven amino acids, we performed a comparative analysis. Of the four linkers, the Motif2 linker contains only one Ala (A), but Motif4,6,8 contain two, and there is a “T” in Motif8, implying greater production of α-helices in Motif8, which may be the reason that the Motif2 protein exhibited the worst function. Likewise, the number of linkers in Motif1,3,5,7 was the same and are 11 amino acids in length, which are similar in properties, leading us make comparisons at some relatively non-conserved sites. In linker positions 7, 8 and 10, there are “R”, “E” and “A” in the Motif1 linker, implying that the Motif1 protein has stronger ability to bind substrates and there is a greater likelihood of producing α-helices.

It follows that the function of an artificial protein is largely related to the characteristic of amino acids contained within the motif, but the amino acids in linker of the repeat unit also play important roles. Here, we propose a scheme (not necessarily to be realized) about designing proteins based on the above studies and analyses. First, we select “motif8”, because it contains more amino acids that can produce α-helices. With regard to linkers, we can consider using “motif1” or “motif8” linkers. There is a greater likelihood of binding to substrates or producing α-helices, respectively. Our results implied that eight artificial proteins with 11-mer repeating motifs were responsible for the ordered structure and chaperone-like function in stress conditions, providing guidance for future investigations as to whether their function is related to the selection of amino acids.

## 4. Materials and Methods

### 4.1. Strains and Plasmids

Bacterial strains and plasmids used in this study are shown in Table 3. *E. coli* BL21(DE3) was used as the host strain to express proteins. The genes, *Motif1*, *Motif2*, *Motif3*, *Motif4*, *Motif5*, *Motif6*, *Motif7* and *Motif8*, were chemically synthesized and inserted into pET28a to form plasmid pET28a–*Motif1*, pET28a–*Motif2*, pET28a–*Motif3*, pET28a–*Motif4*, pET28a–*Motif5*, pET28a–*Motif6*, pET28a–*Motif7*, pET28a–*Motif8* with *Nde* I and *Xho* I restriction sites. The sequences of all vector constructs were verified using Sanger sequencing (Sangon Biotech Co., Ltd., Shanghai, China).

### 4.2. Culture Medium and Conditions

*E. coli* BL21(DE3) cells carrying the corresponding plasmids were cultured in 50 mL flasks containing 20 mL of Luria-Bertani (LB) medium (10 g/L tryptone, 5 g/L yeast extract, and 10 g/L NaCl) with appropriate antibiotics for 12 h at 37 °C and 220 rpm.

### 4.3. Protein Induction and Purification

*E. coli* BL21(DE3) cells carrying the corresponding plasmids were cultured in 500 mL flasks containing 200 mL of LB medium with appropriate antibiotics for 2 h at 37 °C and induced using 0.3 mM IPTG at 16 °C for 12 h. Then, cells expressing proteins were lysed in NTA-0 buffer (300 mM NaCl, 50 mM NaH_2_PO_4_·2H_2_O, 60–100 mL Glycerol, pH 8.0) containing the protease inhibitor and purified by AKTA instrument with 5 mL HisTrap FF Crude prepacked column (Supplementary Figure S1). The concentration of the protein was determined by Brandford [57].

A total of 30 μL of protein was mixed with 70 μL of phosphate buffer, followed by adding 1 mL coomassie brilliant blue. OD_595_ was measured immediately after reaction for 5 min at room temperature. Finally, we obtained the concentration of each protein according to the standard curve.

### 4.4. Circular Dichroism (CD) Spectroscopic Analysis

A total of 0.23 mg/mL protein solution in 5 mM phosphate buffer (pH 7.0) was analyzed with the use of a CD spectropolarimeter (Chirascan, London, UK) with a 0.1 mm path length cuvette at wavelengths of 190–260 nm as previously described in detail [58]. The acquisition parameters were 0.1 nm resolution, 1.0 nm bandwidth, 2 s response and 190–260 nm range. 50% TFE or 50% glycerol were added into the system. Relative secondary structure content of all proteins was analyzed using CDpro software (Figure 2 and Supplementary Table S2).

### 4.5. Diverse Stresses Tolerance in E. coli

*E. coli* recombinant strains (BL21–pET, BL21–HD, BL21–Motif1, BL21–Motif2, BL21–Motif3, BL21–Motif4, BL21–Motif5, BL21–Motif6, BL21–Motif7, BL21–Motif8) were cultured in LB with appropriate antibiotics to OD_600_ ≈ 0.6 at 37 °C and were induced with IPTG at a final optimal concentration of 0.1 mM for 2 h. Subsequently, 1 mL of the cell suspension was pipetted into a new centrifuge tube, after which cells were treated with 10 mM H_2_O_2_ for 15 min, desiccated for 20 days, 0.8 M NaCl for 48 h and freeze-thawed 3 times for 20 min each time, as previously described [59]. Different serial dilutions of these cells were plated onto LB agar plates and incubated at 37 °C overnight before colonies were observed and calculated. The survival rate was expressed as the percentage of the number of colonies in the treated samples compared with those in untreated controls. All experiments were performed at least three times.

### 4.6. LDH/MDH Enzymatic Activity Assay In Vitro

The standard LDH/MDH enzymatic activity was measured as previously described [60]. In total, LDH and additives were diluted in 25 mM Tris–HCl (pH 7.5) to a final concentration of 8.3 μg/mL. For the oxidation treatment, the enzyme mixture was used at 10/20 mM H_2_O_2_ for 15 min. For the desiccation treatment, the enzyme mixture was left in a SpeedVac using centrifugal for 4 h per time and then rehydrated in the same volume of 25 mM Tris–HCl (pH 7.5). For the repeated freeze–thawing treatment, the enzyme mixture was placed in the freezer at −80 °C for 20 min. The reaction was initiated by the addition of oxaloacetic acid (OAA). MDH activity was assessed in the same manner. The reaction was initiated by the addition of pyruvic acid (PYR). The enzymes with a final concentration of 1 mg/mL and proteins were diluted in 50 mM potassium phosphate buffer (pH 7.2). Stress treatments were performed with the same method of LDH.

MDH and LDH activities were assessed by measuring the initial rates of decrease in absorbance at 340 nm in 1 min due to the conversion of NADH into NAD^+^.

### 4.7. Total Antioxidant Capacity, Catalase Activity and MDA Level Analysis

Cells were cultivated to OD_600_ ≈ 0.6 at 37 °C, treated with 20 mM H_2_O_2_ at the final concentration and collected for ultrasonication. The supernatant was transferred to a new tube for subsequent enzyme activity experiments. PBS was added to adjust the volume of each sample to the same protein concentration. The total antioxidant capacity was measured with the FRAP method by using the kit (S0116, Beyotime, Shanghai, China) [61]. The catalase activity was measured with chromogenic reaction by using the kit (S0051, Beyotime, Shanghai, China) [62]. The MDA level was measured with chromogenic reaction using the kit (S0131, Beyotime, Shanghai, China) [63].

## 5. Conclusions

In conclusion, with the aid of synthetic biology, we identified eight novel artificial proteins with different tandem repeats of the same 11-mer motif. The artificial proteins showed highly ordered secondary structures under normal conditions. These proteins enhanced *E. coli* tolerance to different stresses and could protect enzyme activity in vitro. In addition, the recombinant *E*. *coli* containing artificial proteins had higher activities of total antioxidant capacity and less MDA production in vivo than that of the control group after oxidation treatment. Most importantly, the amino acids, different from other motifs, promoted great performance of Motif1 and Motif8, which inspires us to synthesize proteins according to the rules of G3LEA’s motif with optimal amino acids to create artificial elements that participate in abiotic stress response in a chaperone-like manner.

## Figures and Tables

**Table 1:** The levels of CAT activities under oxidative stress.

Name	0 mM H_2_O_2_	20 mM H_2_O_2_
BL21–pET	5.3039	3.0684
BL21–HD	5.2450	4.6380
BL21–Motif1	5.2406	4.3771
BL21–Motif2	5.3995	3.7208
BL21–Motif3	5.5459	3.5968
BL21–Motif4	5.4094	3.4982
BL21–Motif5	5.2206	3.5353
BL21–Motif6	5.3817	3.4173
BL21–Motif7	5.1463	3.8108
BL21–Motif8	5.5093	4.2346

**Table 2:** The content of MDA under oxidative stress.

Name	0 mM H_2_O_2_	20 mM H_2_O_2_
BL21–pET	53.5783	146.0222
BL21–HD	51.5800	60.2700
BL21–Motif1	55.6324	66.2613
BL21–Motif2	53.8313	116.2587
BL21–Motif3	52.9995	87.9578
BL21–Motif4	53.2835	82.7497
BL21–Motif5	55.7432	85.7624
BL21–Motif6	51.1800	88.9068
BL21–Motif7	52.6298	73.3786
BL21–Motif8	50.9233	63.0585

**Table 3:** List of strains and plasmids used in this study.

Strains/Plasmids	Relevant Genotype or Description	Source
Strains		
BL21(DE3)	An *E. coli* strain with T7 RNA polymerase and without Lon protease	Vazyme biotech co.
BL21–pET	The strain containing plasmid pET28a	This study
BL21–HD	The strain containing plasmid 28a–*HD*	This study
BL21–Motif1	The strain containing plasmid 28a–*Motif1*	This study
BL21–Motif2	The strain containing plasmid 28a–*Motif2*	This study
BL21–Motif3	The strain containing plasmid 28a–*Motif3*	This study
BL21–Motif4	The strain containing plasmid 28a–*Motif4*	This study
BL21–Motif5	The strain containing plasmid 28a–*Motif5*	This study
BL21–Motif6	The strain containing plasmid 28a–*Motif6*	This study
BL21–Motif7	The strain containing plasmid 28a–*Motif7*	This study
BL21–Motif8	The strain containing plasmid 28a–*Motif8*	This study
Plasmids		
pET28a	carrying N/C–terminal His tag	Laboratory stock
28a–*HD*	pET28a–derived plasmid carrying the *HD* gene	This study
28a–*Motif1*	pET28a–derived plasmid carrying the *Motif1* gene	This study
28a–*Motif2*	pET28a–derived plasmid carrying the *Motif2* gene	This study
28a–*Motif3*	pET28a–derived plasmid carrying the *Motif3* gene	This study
28a–*Motif4*	pET28a–derived plasmid carrying the *Motif4* gene	This study
28a–*Motif5*	pET28a–derived plasmid carrying the *Motif5* gene	This study
28a–*Motif6*	pET28a–derived plasmid carrying the *Motif6* gene	This study
28a–*Motif7*	pET28a–derived plasmid carrying the *Motif7* gene	This study
28a–*Motif8*	pET28a–derived plasmid carrying the *Motif8* gene	This study

## Data Availability

All data underlying the results are included as part of the published article and its Supplementary Materials.

## Supplementary Materials

The following supporting information can be downloaded at: https://www.mdpi.com/article/10.3390/antiox12061147/s1.
