# Supplementary material for: Artificial Proteins Designed from G3LEA Contribute to Enhancement of Oxidation Tolerance in E. coli in a Chaperone-like Manner
Source: Antioxidants (Basel). 2023 May 24;12(6):1147. doi: 10.3390/antiox12061147 (PMC10295645; doi:10.3390/antiox12061147)
Supplement: Supplementary file 1 [file antioxidants-12-01147-s001.zip › antioxidants-2290383-supplementary.pdf]

Supplementary Materials

Artificial Proteins Designed from G3LEA Contribute to Enhancement of Oxidation Tolerance in *E. coli* in a Chaperone-like Manner

Jiahui Han [1], Shijie Jiang [2], Zhengfu Zhou [1], Min Lin [1] and Jin Wang [1,*]

[1] Key Laboratory of Agricultural Microbiome (MARA), Biotechnology Research Institute, Chinese Academy of Agricultural Sciences, Beijing 100081, China
[2] School of Life Science and Engineering, Southwest University of Science and Technology, Mianyang 621010, China
* Correspondence: wangjin@caas.cn

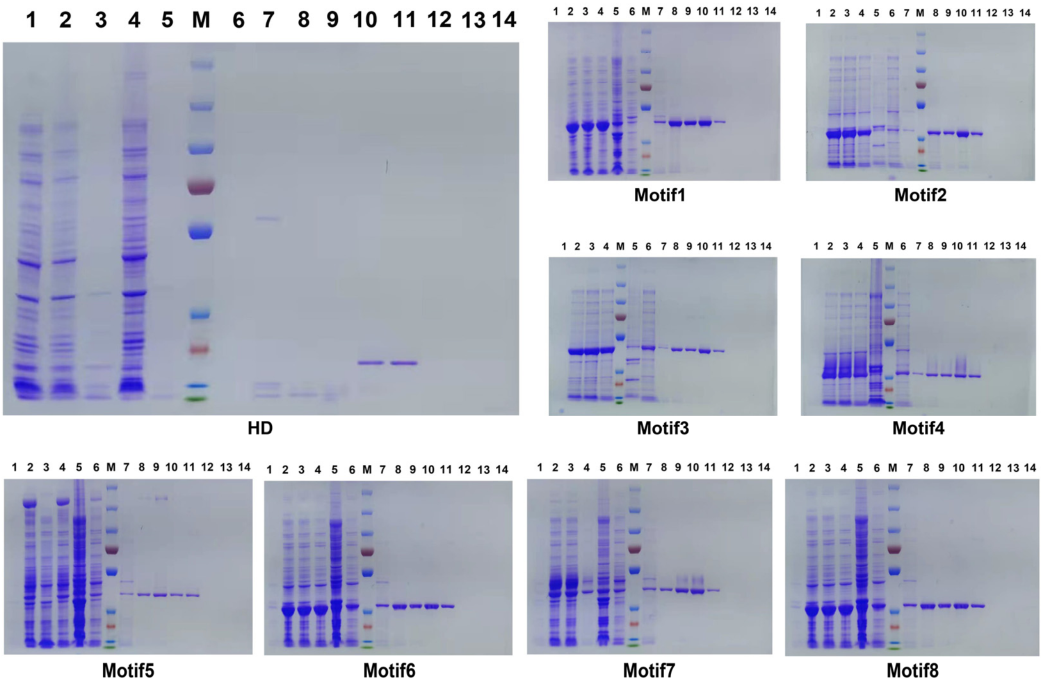

Figure S1. SDS-PAGE analysis of nine purified proteins. Lane 1, cells, lane 2, broken liquid, lane 3, supernatant after ultrasonication, lane 4, sediment, lane 5, penetration, lane 6, elution with 0 mM imidazole, lane 7, elution with 10 mM imidazole, lane 8, elution with 20 mM imidazole, lane 9, elution with 50 mM imidazole, lane 10, elution with 80 mM imidazole, lane 11, elution with 100 mM imidazole, lane 12, elution with 150 mM imidazole, lane 13, elution with 200 mM imidazole, lane 14, elution with 300 mM imidazole. Lane M, molecular weight marker (kDa).

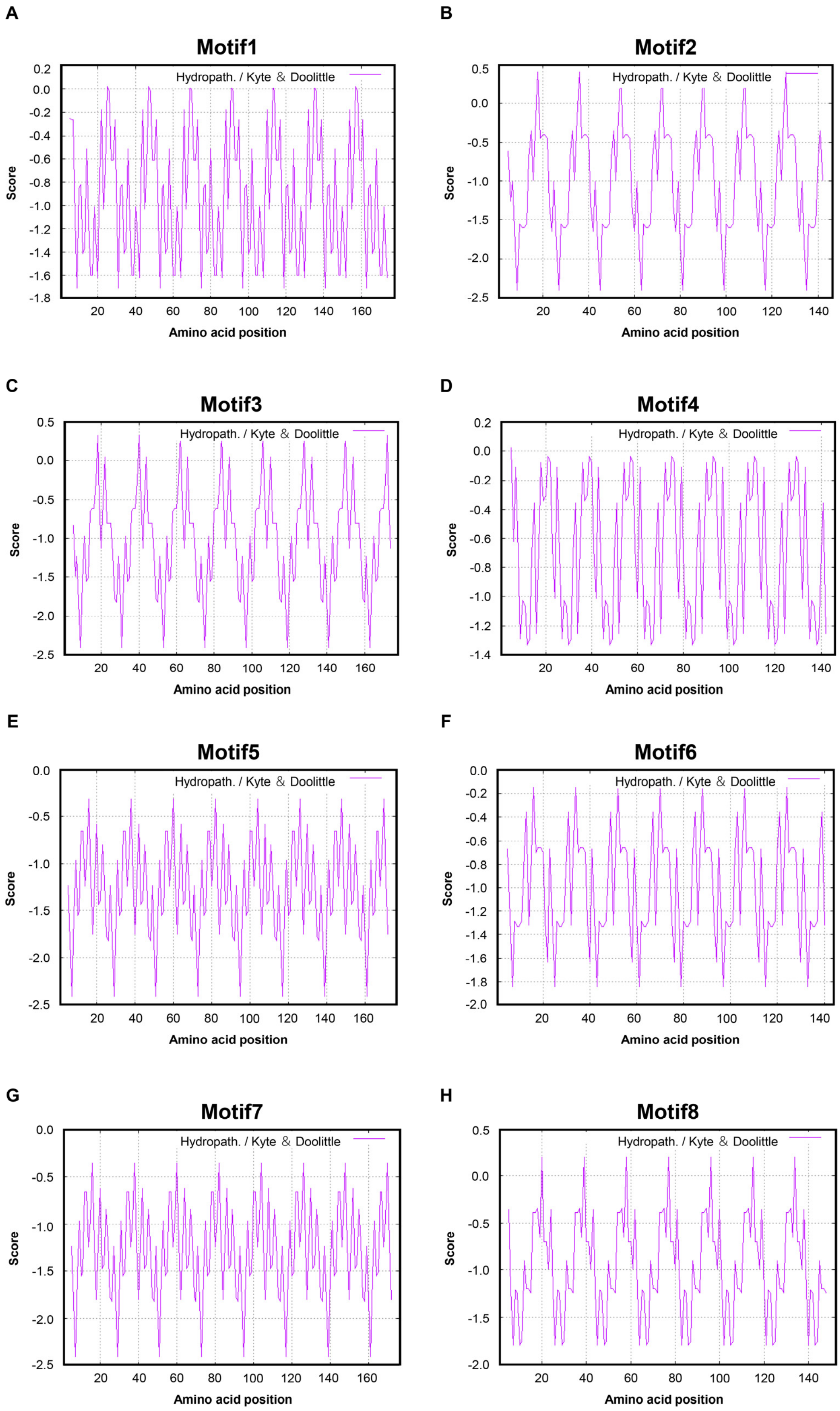

Figure S2. Hydropathic index plot of the eight proteins amino acid sequences analyzed by using the Kyte-Doolittle algorithm. Regions with a hydropathy score below zero are hydrophilic. A-H represent the prediction of the hydrophobicity of Motif1-8 proteins.

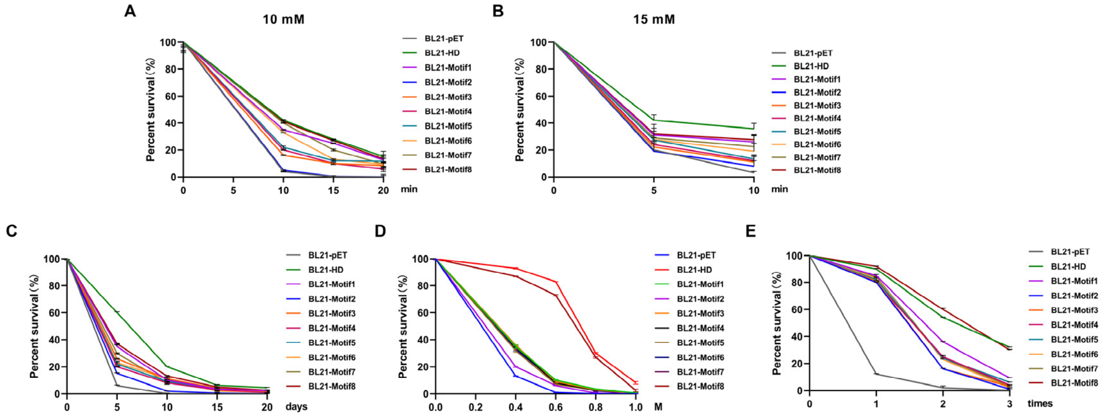

Figure S3. Cell survival under different stress conditions. Survival of recombinant *E. coli* under different conditions of 10 mM H_2O_2 (A), 15 mM H_2O_2 (B), desiccation (C), NaCl (D) and freeze-thawing (E).

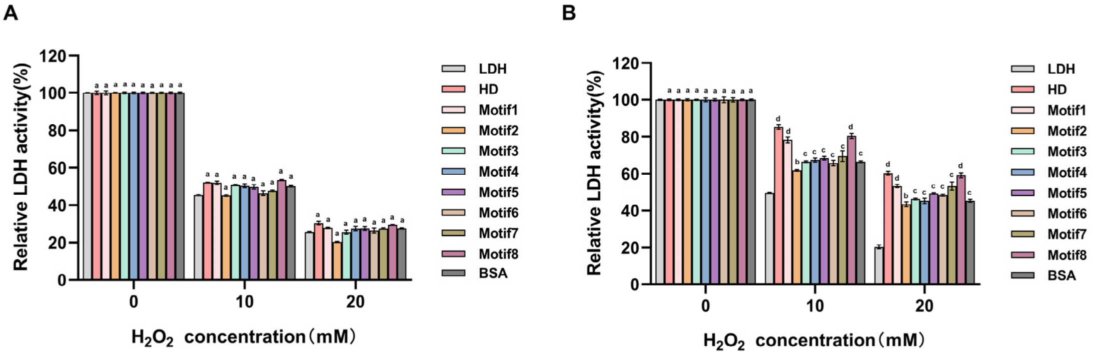

Figure S4. The relative activity of LDH under the oxidation. The LDH activity with proteins (proteins: LDH=1:1) (A) and (proteins : LDH=5:1) (B).

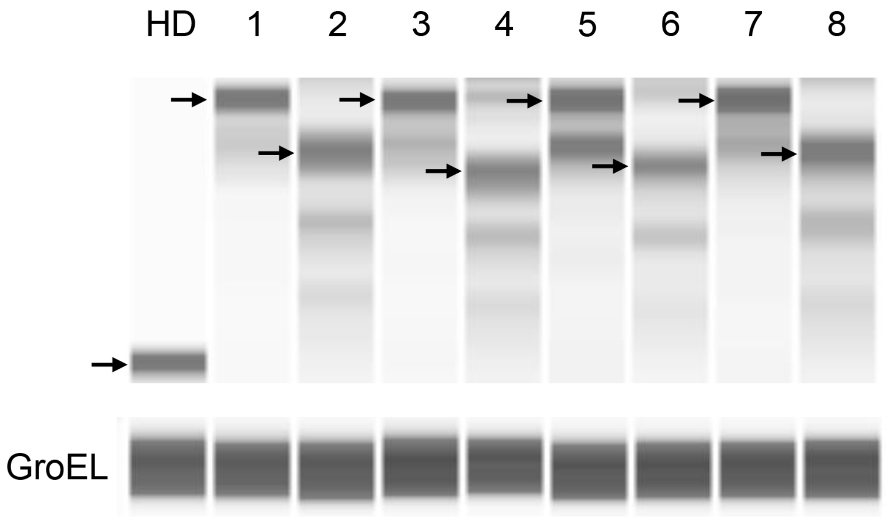

Figure S5. The expression level of different artificial proteins. 1-8 represent Motif1-8. The black arrows point to the bands of target proteins.

Table S1. The composition of each artificial protein analyzed by ProtParam.

Name of artificial proteins	Amino acids of full length (aa)	Grand average of hydropathicity (GRAVY)	Instability index	Total number of negatively charged residues (Asp + Glu)	Total number of positively charged residues (Arg + Lys)
HD	160	-0.991	17.00	28	27
Motif1	176	-0.918	5.04	32	24
Motif2	144	-1.000	0.75	32	32
Motif3	176	-1.009	15.87	24	16
Motif4	144	-0.683	3.28	24	32
Motif5	176	-1.268	25.33	24	24
Motif6	144	-0.994	15.58	32	32
Motif7	176	-1.286	39.16	32	24
Motif8	144	-0.942	32.32	24	24

Note: If instability index is <40, the protein is stable, if not, the protein is unstable.

Table S2. Secondary structure content in nine proteins was obtained by far-UV CD spectrometry and calculated with CDNN program.

Treatment	Protein	α-helix (%)	β-sheet (%)	turn (%)	random coil (%)
Phosphate buffer	HD	12.0	11.0	17.0	60.0
	Motif1	97.4	0.2	2.3	0.1
	Motif2	87.2	3.6	9.0	0.2
	Motif3	90.0	6.0	4.0	0.0
	Motif4	80.3	4.6	14.2	0.9
	Motif5	96.0	0.3	3.6	0.1
	Motif6	85.3	1.7	11.8	1.2
	Motif7	83.4	2.4	12.7	1.5
	Motif8	95.6	0.7	3.6	0.1
50% TFE	Protein	α-helix (%)	β-sheet (%)	turn (%)	random coil (%)
	HD	95.0	1.0	3.0	1.0
	Motif1	99.8	0.1	0.0	0.1
	Motif2	95.2	0.1	4.6	0.1
	Motif3	95.6	0.1	4.2	0.1
	Motif4	96.4	0.1	3.2	0.3
	Motif5	95.8	0.1	4.0	0.1
	Motif6	95.5	0.1	4.3	0.1
	Motif7	96.0	0.1	3.8	0.1
	Motif8	99.6	0.1	0.1	0.2
50% Glycerol	Protein	α-helix (%)	β-sheet (%)	turn (%)	random coil (%)
	HD	92.0	1.0	6.0	1.0
	Motif1	98.6	0.1	1.2	0.1
	Motif2	93.5	0.0	6.5	0.0
	Motif3	94.9	0.1	4.9	0.1
	Motif4	97.6	0.2	2.0	0.2
	Motif5	95.1	0.1	4.7	0.1
	Motfi6	96.8	0.3	2.5	0.4
	Motif7	97.2	0.3	2.1	0.4
	Motif8	98.0	0.5	1.1	0.4
